# Fatigue Behavior of Concrete Beam with Prestressed Near-Surface Mounted CFRP Reinforcement According to the Strength and Developed Length

**DOI:** 10.3390/ma12010051

**Published:** 2018-12-24

**Authors:** Hee Beom Park, Jong-Sup Park, Jae-Yoon Kang, Woo-Tai Jung

**Affiliations:** Structural Engineering Research Institute, Korea Institute of Civil Engineering and Building Technology, Goyang 10223, Korea; heebeompark@kict.re.kr (H.B.P.); jspark1@kict.re.kr (J.-S.P.); jykang@kict.re.kr (J.-Y.K.)

**Keywords:** CFRP, fatigue, prestressed near-surface mounted reinforcement (NSMR), strengthening, tendon

## Abstract

The prestressed near-surface mounted reinforcement (NSMR) using Fiber Reinforced Polymer (FRP) was developed to improve the load bearing capacity of ageing or degraded concrete structures. The NSMR using FRP was the subject of numerous studies of which a mere portion was dedicated to the long-term behavior under fatigue loading. Accordingly, the present study intends to examine the fatigue performance of the NSMR applying the anchoring system developed by Korea Institute of Construction and Building Technology (KICT). To that goal, fatigue test is performed on 6.4 m reinforced concrete beams fabricated with various concrete strengths and developed lengths of the Carbon Fiber Reinforced Polymer (CFRP) tendon. The test results reveal that the difference in the concrete strength and in the developed length of the CFRP tendon has insignificant effect on the strengthening performance. It is concluded that the accumulation of fatigue loading, the concrete strength and the developed length of the tendon will not affect significantly the strengthening performance given that sufficient strengthening is secured.

## 1. Introduction

Prestressed concrete (PSC) eases the control of the deflection and cracks in concrete structures by reducing the tensile stress through the introduction of a compressive force by means of tendons embedded in the tension zone of the structure. Ageing bridges may experience loss of their function and performance that should be recovered or improved by strengthening the structure. The strengthening of PSC girder bridge is achieved by enlarging the girder, external prestressing, carbon fiber bonding or steel plate bonding. The external bonding reinforcement (EBR) methods were applied to improve the performance by bonding the reinforcement on the tensile zone of the concrete member using an adhesive. This reinforcement took first the form of a plate made of steel that started to be replaced by Fiber Reinforced Polymer (FRP) since the mid of 1980s.

The near-surface mounted reinforcement (NSMR) resembles the above mentioned external bonding reinforcement but embeds the FRP plate or bar in a groove cut with a definite depth in the concrete surface. De Lorenzis et al. [1] reported that the superior resistance to bond failure provided by NSMR compared to EBR brought higher improvement of the performance and that the embedment of the tendon in concrete could prevent its damage due fire or vehicle impact. Moreover, these authors also reported that additional resistance to physical wearing caused by the tires of passing vehicles can be expected when NSMR is applied to strengthen the negative moment zone of the deck.

In view of the efficiency of FRP, both NSMR and EBR cannot exploit fully the maximum performance of FRP due to the premature occurrence of debonding at the reinforcement-concrete interface. As passive strengthening methods, their effect appears only under the application of additional live loads without clear improvement of the serviceability in term of cracking and deflection recovery. Accordingly, numerous researchers attempted the prestressed NSMR achieving the synergy of both NSMR and prestressing [2]. For the prestressed NSMR to be realizable, the most important matter is the development of appropriate anchor and tensioning system enabling to apply the prestress force using FRP. El-Hacha and Soudki [2] employed temporary clamp-anchors and pre-tensioning or end-supported tensioning devices. Korea Institute of Construction and Building Technology (KICT) developed a tensioning device using anchors fixed to the concrete beam as tensile reaction table [3].

In particular, FRP is checked to apply in many sites because of strong points such as high strength, light weight, chemical resistance and need to secure reliability through real size test because of low applications, low construction cases, problems with design criteria [4,5,6,7,8].

Despite of the numerous studies related to NSMR using FRP, a very few of them studied the long-term behavior under fatigue loading [9]. The repeated action of the fatigue loading is likely to degrade the quality of FRP and concrete and the concrete-FRP-filler bond performance in the structure strengthened by NSMR. A few studies have been published on RC beams strengthened by the NSM FRP system with epoxy adhesive and subjected to flexural fatigue loading [10,11,12,13,14,15].

Accordingly, the present study intends to examine the fatigue behavior of the prestressed NSMR applying the anchoring device developed by KICT. The cumulated effect of the fatigue load on the strengthening performance is examined by means of a series of fatigue tests performed on 6.4 m reinforced concrete beams fabricated with various concrete strengths and developed lengths of the Carbon Fiber Reinforced Polymer (CFRP) tendon. The fatigue tests were conducted in two stages. The first stage applied 2 million loading cycles and the second stage applied static loading until failure of the specimens to measure the residual strength after the accumulation of fatigue.

## 2. Experimental Program

### 2.1. Test Variables

**Figure 1:**
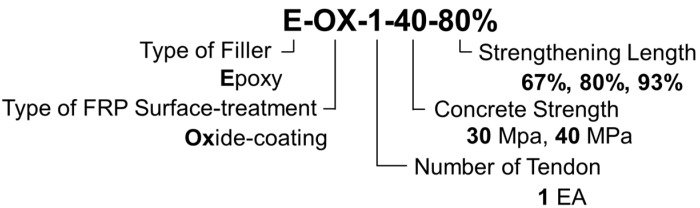
Designation of specimens.

This study intends to examine the fatigue performance of the prestressed NSMR. To that goal, the specimens are designated as shown in Figure 1 with respect to the concrete strength and developed length of the CFRP tendon chosen as test variables. In Figure 1, the first string of character indicates the type of filler with E for epoxy. The second string of character announces the type of surface treatment applied on the FRP tendon with OX for oxide-coating. The third string of character designates the amount of reinforcement with 1 for one line of reinforcement. The fourth string of character stands for the concrete strength with 30 for 30 MPa and 40 for 40 MPa. The fifth string of character indicates the developed length of the CFRP tendon. Specifically, for the span length of 6000 mm, 67% represents 4000 mm and 93% represents 5600 mm with reference to the length of 4800 mm corresponding to about 80% of the span length. Table 1 lists the adopted specimens with their designation according to the test variables. Steel rebars had a modulus of elasticity of 200 GPa and a yielding stress of 400 MPa. Epoxy had a tensile strength of 47 MPa and a bond strength of 9 MPa.

### 2.2. Fabrication of Specimens

**Figure 2:**
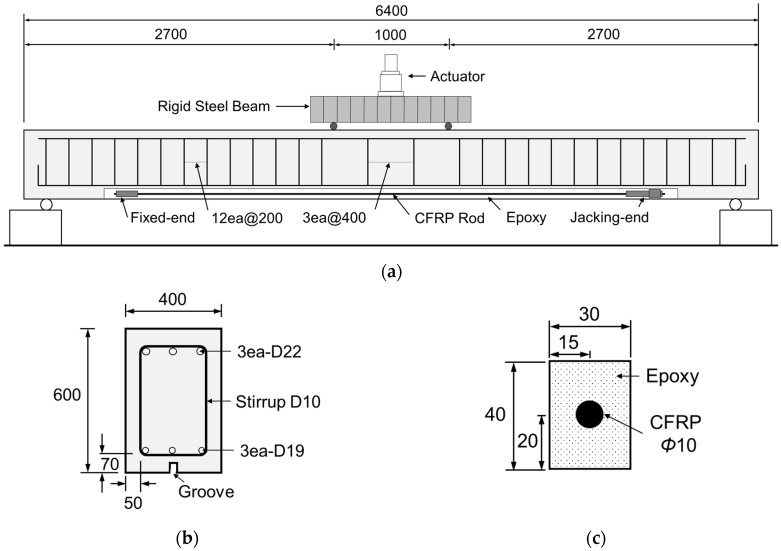
Test setup and beam details (all dimensions are in mm): (**a**) beam dimensions; (**b**) cross section; (**c**) groove.

As shown in Figure 2a, the reinforced concrete specimens were fabricated to have a total length of 6400 mm with a span length of 6000 mm. Figure 2b depicts the reinforcement details of the specimens. Three D19 bars are used as tensile reinforcement and D22 bar is disposed in the compression zone to prevent the occurrence of compressive failure before tensile failure. Moreover, D10 stirrups are installed every 200 mm to provide sufficient resistance to shear. For the prestressed NSMR, grooves must be cut for the anchoring of the FRP tendon to be embedded in concrete. As shown in Figure 2c, the grooves are rectangular with width of 30 mm and depth of 40 mm. The surface is finished by applying epoxy after the tensioning.

### 2.3. Test Setup

The structural tests were executed in two stages. The first stage applied 2 million loading cycles and the second stage applied static loading until failure of the specimens to measure the residual strength after the accumulation of fatigue.

The size of the fatigue load was determined with reference to the stress of the tensile member using the calculation method suggested by ACI, AASHTO and CSA [16,17,18,19]. CSA limits the range of the tensile member stress below 125 MPa, and ACI limits the size of the fatigue load within 80% of the yield strength. Based on these recommendations, Oudah and El-Hacha [20] conducted their experiments with a fatigue load ranging between 0.42f_y_ and 0.7f_y_, where f_y_ is the steel yield stress. Accordingly, the present study adopted fatigue load ranging between 60 kN and 100 kN. In addition, the loading rate was set to 2.0 to 3.0 Hz. The fatigue test conducted using a dynamic actuator with capacity of 1000 kN to apply 2 million loading cycles. Static load of 100 kN was applied after 1, 1000, 5000, 10,000, 100,000, 1 million and 2 million cycles to measure the deflection, strain and cracks and examine the eventual progress of damage according to the accumulation of fatigue.

**Figure 3:**
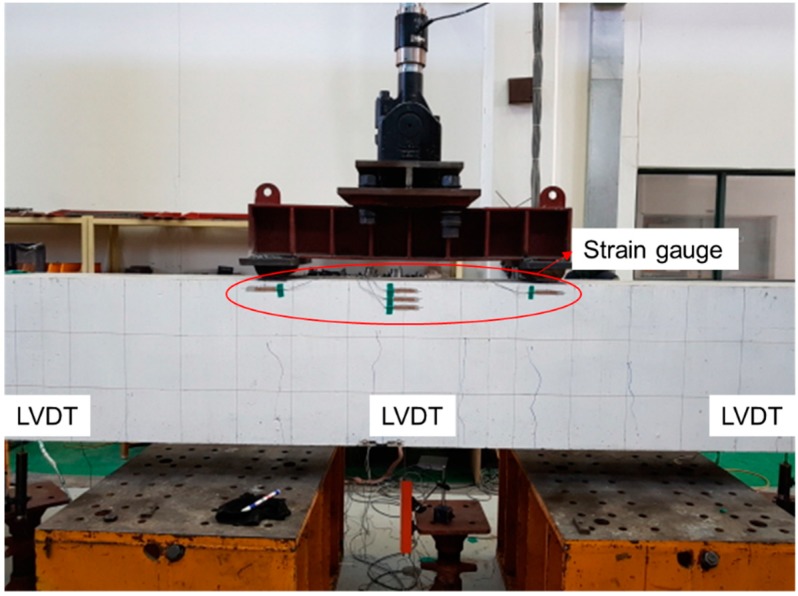
Sensor setup.

Static loading was performed by 4-point loading on all the specimens using static actuators (Korea Institute of Civil Engineering and Building Technology, Goyang-Si, Korea) with capacity of 2000 kN to measure the deflection and strain according to the gradual increase of the load until failure. As shown in Figure 2a, loading was applied identically on all the specimens at the points located 500 mm far from the center to secure a pure flexure section of 1 m. The static loading was conducted through displacement control at speed of 0.03 mm/s for the first 20 mm and at speed of 0.05 mm/s beyond that displacement and until failure. Rebar strain gauges were attached to the upper and lower reinforcements to measure the rebar strain according to the load. Strain gauges were also attached at the center and quarter points of the CFRP tendon to grasp the strain change throughout the tests. As shown in Figure 3, the load-displacement curves were measured by reading directly the load of the actuator and the displacement from a LVDT installed at mid-span of the specimens.

### 2.4. Tensioning

**Figure 4:**
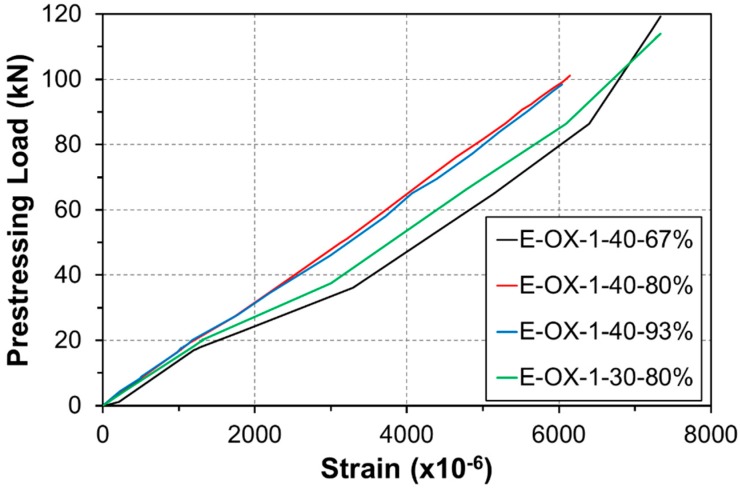
CFRP prestressing load wrt stress-strain curve.

The tendon used in the fabrication of NSM specimens is a CFRP rod with diameter of 10 mm and its mechanical characteristics are arranged in Table 2. The allowable tension force to be introduced in the proposed FRP prestressed NSMR ranges between 40% and 65% of the strength of the FRP tendon [16]. Accordingly, the tension force of 100 kN corresponding to approximately 42% of the tendon strength was applied in the fabrication of the specimens. The tension force was measured by means of the load cell attached to the hydraulic cylinder and verified using the sensors disposed on the FRP tendon. Figure 4 plots the tension force measured the tensioning of the prestressed NSMR. Note that the strain of the CFRP tendon ranged between 6000 × 10^−6^ and 6500 × 10^−6^ during the tensioning work.

## 3. Test Rsults

### 3.1. Fatigue Performance Acording to Concrete Srength

**Figure 5:**
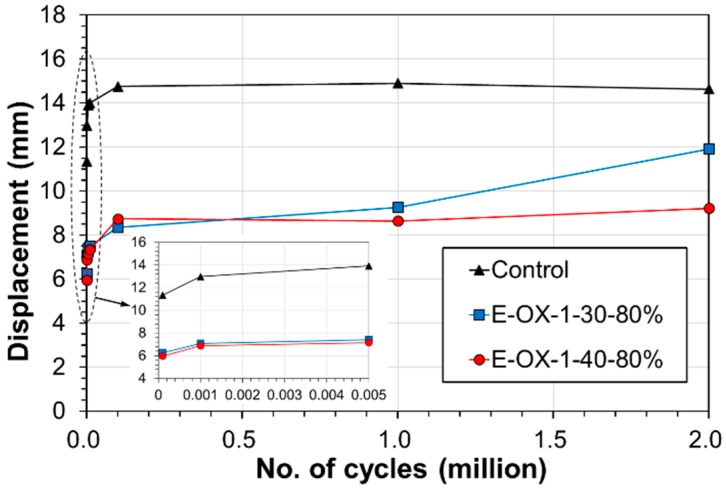
Variation of mid-span deflection during fatigue loading at upper load limit.

Table 3 arranges the values of the deflection at mid-span, the strains at the center of the upper and lower rebars, and the strain change of the CFRP tendon according to strength of the concrete and measured in each specimen under static load of 100 kN after 1, 1000, 2 million fatigue loading cycles. The increase of the deflection at mid-span following the accumulation of fatigue is seen to be provoked by the concrete creep, the degradation of the material bonding the tendon and concrete, and the plastic deformation of the CFRP tendon [21,22]. As shown in Figure 5, the deflection experienced the largest increase rate below 1000 loading cycles and increased gradually until 100,000 cycles to remain practically constant until 2 million cycles.

**Figure 6:**
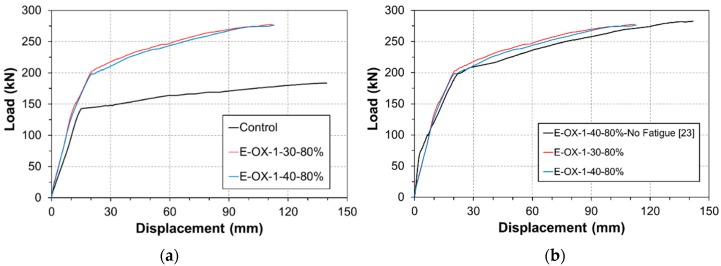
Load-displacement relationships (Concrete strength): (**a**) vs. control; (**b**) vs. no fatigue.

The residual strength can be measured using the static loading results listed in Table 4. Figure 6a plots the load-deflection curves measured at mid-span. The comparison of the residual strength with respect to the concrete strength reveals that, compared to Control, specimen E-OX-1-30-80% developed yield strength larger by 42% and ultimate strength larger by 51%. Similar observation can also be done for specimen E-OX-1-40-80%.

Figure 6b shows the residual strength of the specimens after fatigue loading. The results are seen to be nearly identical to those obtained in a previous work [23] in which static loading was applied without preliminary fatigue loading. When static loading is applied without accumulation of fatigue, the load-deflection curve presents three distinct behaviors that are the crack behavior, the yield behavior and the fatigue behavior. However, the load-deflection curve of the specimen which experienced fatigue loading presents two behaviors that are the yield behavior and the fatigue behavior without crack behavior since the specimen has already cracked. This indicates that damage or loss of the performance did practically not occur following the accumulation of fatigue. Moreover, there was no significant difference in the performance developed by the specimens with concrete strength of 30 MPa and 40 MPa. This means that the strengthening effect is not particularly influenced by the accumulation of fatigue nor the concrete strength if sufficient strengthening is secured.

**Figure 7:**
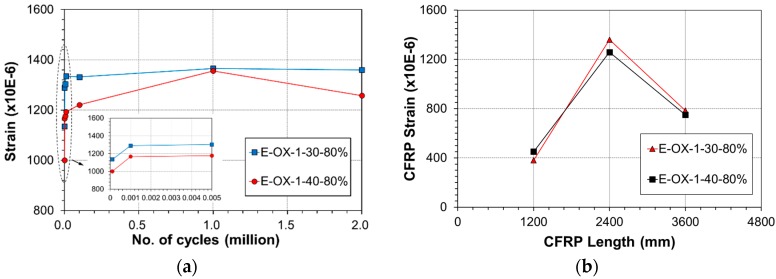
Variation of CFRP strain during fatigue loading at upper load limit: (**a**) mid span; (**b**) location.

Figure 7 plots the strains measured at the center and quarter lengths of the CFRP tendon under the upper load according to the accumulation of fatigue loading cycle. As shown in Figure 7a, similarly to the deflection, the strain exhibited its largest increase rate below 1000 loading cycles and increased gradually until 100,000 cycles to remain nearly unchanged until 2 million cycles. The strain at early fatigue would have decreased in occurrence of slip between the epoxy and the tendon [24] but such behavior cannot be observed here and resembles that of the deflection, which indicates that bond slip did not occur. Recalling that the CFRP tendon is 4800 mm-long, the strains plotted in Figure 7b were measured at the center (2400 mm) and quarter lengths (1200 mm and 3600 mm). In the graph, the horizontal axis represents the position along the length of the CFRP tendon and the vertical axis represents the strain at upper load after 2 million cycles of fatigue loading. The increase of the strain at the center of the tendon can be clearly distinguished with the increase of the load because the CFRP tendon-concrete bond performance is secured by the epoxy filler. This indicates the absence of bond slip in the anchored ends according to the accumulation of fatigue.

**Figure 8:**
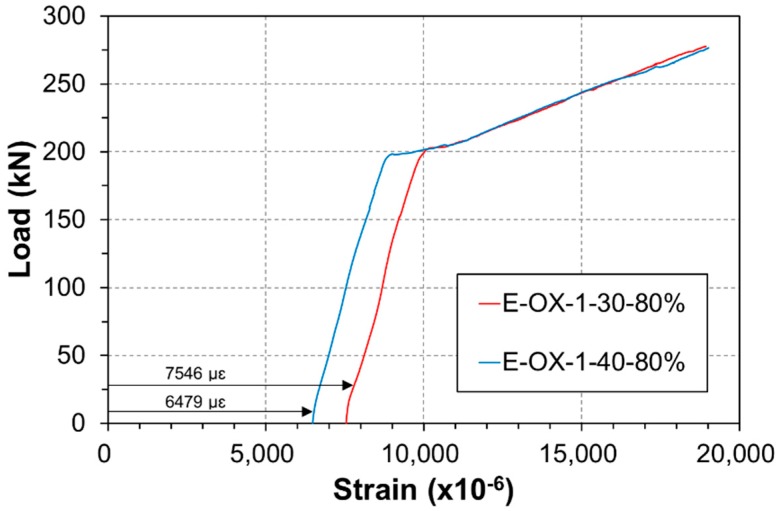
Load-strain relationship at CFRP (Concrete strength).

Figure 8 plots the strain of the tendon under static loading. The indicated strain gathers the strain developed during tensioning and the strain developed along the accumulation of fatigue. Both specimens are seen to show similar strains and behavior at failure of the tendon. In addition, the tendon reached its tensile performance before rupture under the loading applied after the compressive failure of concrete. Consequently, the accumulation of the fatigue load appears to have a poor effect on the loss of the performance or tension force of the CFRP tendon.

**Figure 9:**
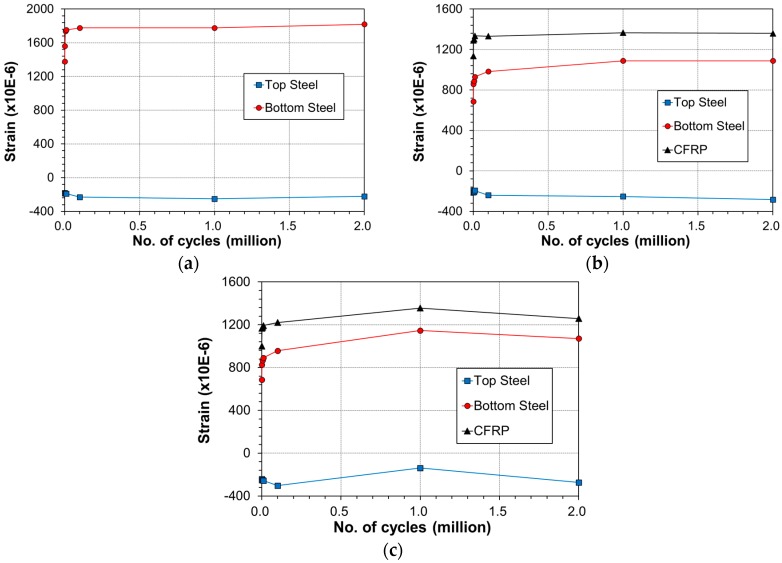
Variation of strain across the beam at mid-span with number of cycles: (**a**) control; (**b**) E-OX-1-30-80%; (**c**) E-OX-1-40-80%.

Figure 9 describes the variation of the strain at mid-span of the beam at upper load according to the accumulation of fatigue loading. The strain in all the specimens experienced steep increase from 1 cycle to 1000 cycles and stabilized gradually until 2 million cycles. In some case, the strain remained unchanged or decreased beyond 1 million cycles.

**Figure 10:**
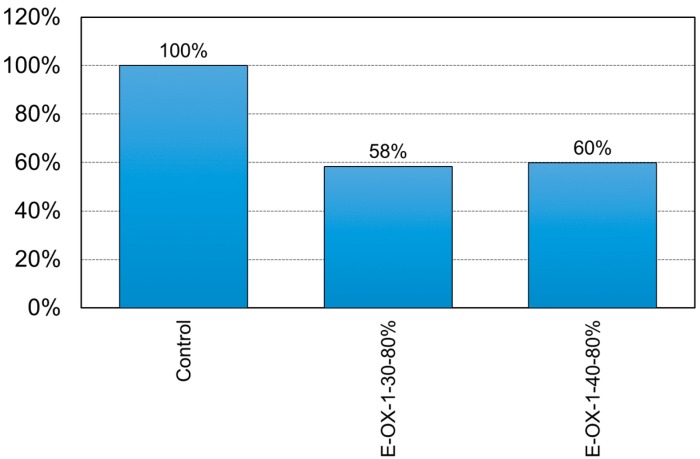
Ductility of NSM specimens (Concrete strength).

The ductility of the specimens with respect to the concrete strength is shown in Figure 10. Here, the ductility is defined as the ratio of the deflection at yielding to that at failure. The ductility of the prestressed NSMR specimens tends to decrease compared to that before strengthening. A previous study reported that the ductility tended to reduce by about 45% when prestressed NSMR is achieved using a CFRP tendon with high bond strength [18]. This is confirmed here with a decrease of the ductility by 42% for specimen E-OX-1-30-80% and by 40% for specimen E-OX-1-40-80% compared to Control and, indicates that the accumulation of fatigue loading does not affect significantly the ductility.

### 3.2. Fatigue Performance According to Developed Length of CFRP Tendon

**Figure 11:**
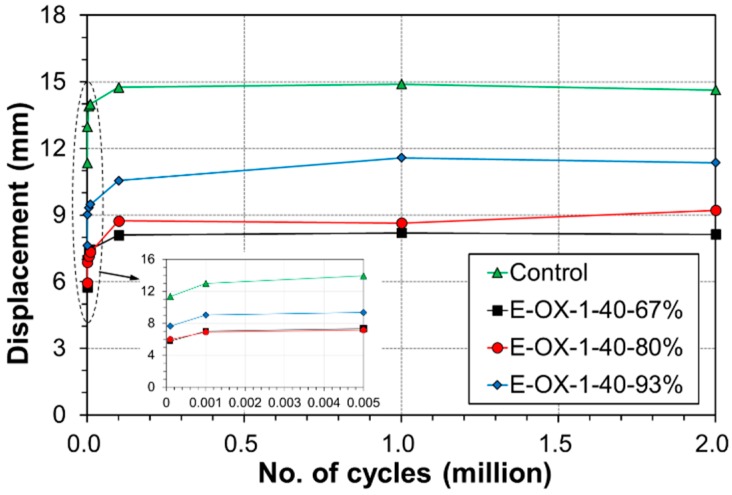
Variation of mid-span deflection during fatigue loading at upper load limit.

Table 5 arranges the deflection at mid-span, the strain in the upper and lower reinforcements at mid-span and the strain change at the center of the CFRP tendon measured under static load of 100 kN after 1, 1000 and 2 million fatigue cycles and according to the developed length of the CFRP tendon. As shown in Figure 11, the deflection exhibited the largest increase rate below 1000 cycles, increased gradually until 100,000 cycles and stabilized until 2 million cycles. Compared to Control, the deflection under upper load after 2 million cycles was smaller by 44% for specimen E-OX-1-40-67%, by 37% for specimen E-OX-1-40-80% and by 22% for specimen E-OX-1-40-93%.

**Figure 12:**
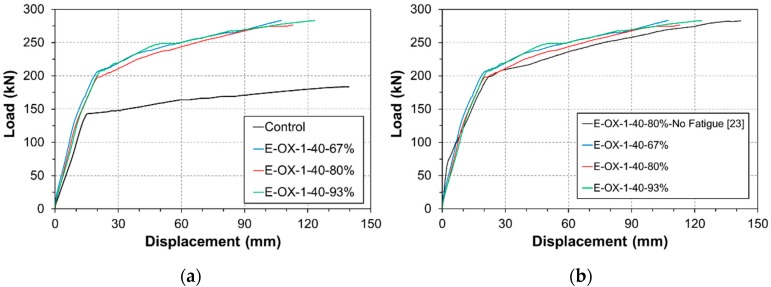
Load-displacement relationships (Bond length): (**a**) vs. control; (**b**) vs. no fatigue.

**Figure 13:**
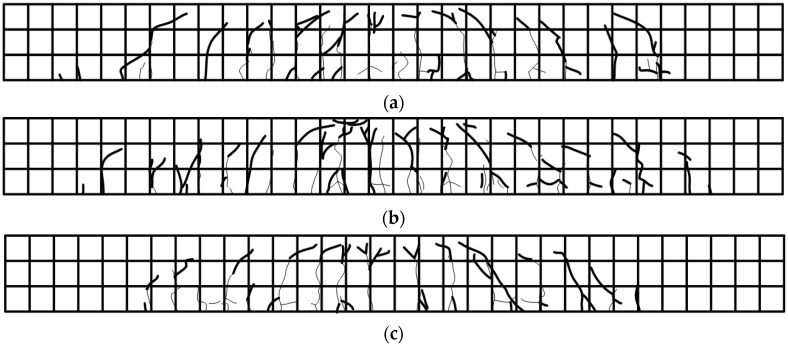
Crack map: (**a**) E-OX-1-40-67%; (**b**) E-OX-1-40-80%; (**c**) E-OX-1-40-93%.

Table 6 arranges the results of the static loading test for the residual strength with respect to the developed length of the CFRP tendon. The load-deflection curves at mid-span of the beam members plotted in Figure 12a show that, compared to Control, specimen E-OX-1-40-67% developed yield load larger by 45% and ultimate load larger by 54% and that specimen E-OX-1-40-80% also developed comparable residual strength. As shown in Figure 12b, the residual strength after fatigue loading is practically identical to that developed by specimens which were subjected only to static loading. This indicates that the accumulation of fatigue does not provoke any damage nor performance loss of the prestressed NSMR specimens. In addition, the insignificance of the change in the performance developed by the specimens with developed lengths of 67%, 80% and 93% of the CFRP tendon demonstrates that the developed length of the tendon has practically no effect on the strengthening performance given that appropriate tensioning has been secured. However, shear failure would occur due to the increase of inclined cracks if the anchors are installed outside the effective depth d [3]. This is the case for specimen E-OX-1-40-67% in which the anchors are disposed outside the effective depth d and inclined cracks are seen to occur around the anchor as shown in Figure 13. Following, an appropriate and sufficient developed length should be secured to prevent the anchor be installed outside the effective depth d.

**Figure 14:**
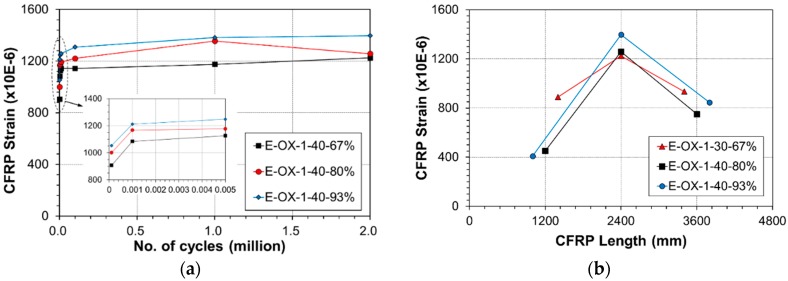
Variation of CFRP strain during fatigue loading at upper load limit: (**a**) mid span; (**b**) location.

Figure 14a plots the strains measured at the center and quarter lengths of the CFRP tendon under the upper load according to the accumulation of fatigue loading. Similarly to the deflection behavior, the strain exhibited the largest increase rate below 1000 cycles, increased gradually until 100,000 cycles and stabilized until 2 million cycles. As mentioned above, this indicates that bond slip did not occur at the epoxy-tendon interface. The strains were measured at lengths of 4000 mm, 4800 mm and 5600 mm along the tendon with reference to the center at the same positions listed in Table 7. Figure 14b plots the strains at upper load measured after 2 million cycles. Here also, it appears that bond slip did not occur at the anchored ends with respect to the length of the tendon.

**Figure 15:**
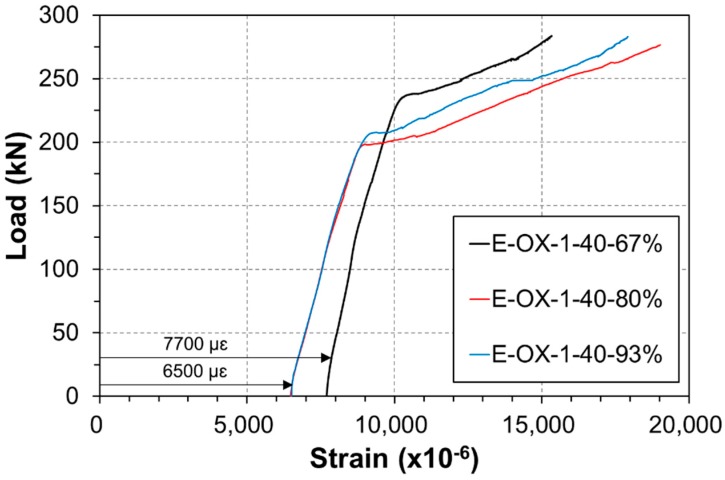
Load-strain relationship at CFRP (Bond length).

Figure 15 presents the strain of the tendon according to the static load. The indicated strains include the strain developed at tensioning and the strain caused by the accumulated fatigue loading. All the three specimens exhibit similar behavior and strain at failure. Moreover, the tendon reached its tensile performance before rupture under the loading applied after the compressive failure of concrete. Accordingly, the accumulation of the fatigue load appears to have poor effect on the loss of the performance like the tension force of the CFRP tendon.

**Figure 16:**
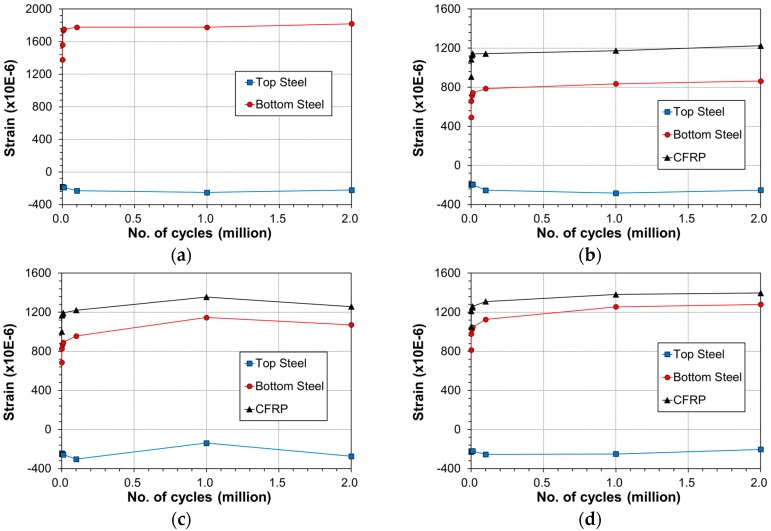
Variation of strain across the beam at mid-span with number of cycles: (**a**) control; (**b**) E-OX-1-40-67%; (**c**) E-OX-1-40-80%; (**d**) E-OX-1-40-93%.

Figure 16 describes the steel and CFRP strain variation at mid-span of the beam members under the upper load according to the accumulation of fatigue loading. The strain in all the specimens is seen to experience the largest increase rate below 1000 cycles, to increase gradually until 100,000 cycles and to stabilize until 2 million cycles. In some case, the strain remained unchanged or decreased beyond 1 million cycles.

**Figure 17:**
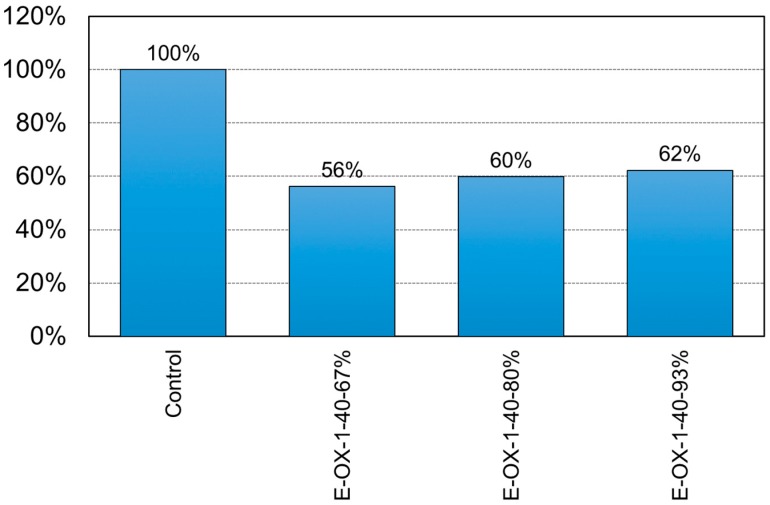
Ductility of NSM specimens (Bond length).

Figure 17 shows the ductility of the specimens with respect to the developed length of the CFRP tendon. The ductility appears to have reduced by 44% for specimen E-OX-1-40-67%, by 40% for specimen E-OX-1-40-80% and by 38% for specimen E-OX-1-93% compared to Control. In other words, the ductility increased with longer developed length of the tendon. Therefore, longer developed length of the tendon seems to be favorable for securing stable ductile behavior. Moreover, since the observed ductility complies with that reported in a previous study [25], it can be stated that the accumulation of fatigue loading has no particular effect on the ductility of the prestressed NSMR specimens with respect to the developed length.

## 4. Conclusions

This study examined the strengthening performance of the prestressed NSMR according to the accumulation of fatigue loading. To that goal, fatigue test was performed on 6.4 m reinforced concrete beams fabricated with various concrete strengths and developed lengths of the CFRP tendon. The fatigue tests were conducted in two stages. The first stage applied 2 million loading cycles and the second stage applied static loading until failure of the specimens to measure the residual strength after the accumulation of fatigue. The following conclusions can be derived.

- The observation of the fatigue behavior with respect to the concrete strength revealed that the deflection exhibited the largest increase rate below 1000 cycles, increased gradually until 100,000 cycles and stabilized until 2 million cycles. The strain measured at the center of the CFRP tendon showed also similar tendency according to the accumulation of fatigue cycles. Moreover, the same tendency was also observed for the fatigue behavior according to the developed length of the tendon.
- The analysis of the strain developed during tensioning and the strain caused by the accumulation of fatigue revealed that all the specimens presented similar behavior and strain at the rupture of the tendon. In addition, the tendon could reach its tensile performance before rupture under the loading applied after the compressive failure of concrete. This indicated that the accumulation of the fatigue load had poor effect on the loss of the performance like the tension force of the CFRP tendon.
- The results of the static loading test with respect to the concrete strength showed that the accumulation of the fatigue load did not provoke damage nor performance loss of the prestressed NSMR specimens. Moreover, the test results of the specimens with concrete strengths of 30 MPa and 40 MPa appeared to be practically identical to those of a previous experiment without fatigue load accumulation. This indicated that the strengthening performance of the prestressed NSMR is insensitive to the accumulation of fatigue loading and the concrete strength given that sufficient strengthening is secured.
- The insignificance of the change in the performance provided by the specimens with developed lengths of 67%, 80% and 93% of the CFRP tendon demonstrated that the developed length of the tendon has practically no effect on the strengthening performance given that appropriate tensioning has been secured. However, an appropriate and sufficient developed length should be secured to prevent the anchor be installed outside the effective depth d in which case shear failure would occur due to the increase of inclined cracks.
- The analysis of the ductility with respect to the concrete strength and the developed length of the tendon provided results complying with those of previous research. All the specimens exhibited nearly the same ductility according to the concrete strength and the ductility appeared to increase with longer developed length of the tendon. Accordingly, adopting longer developed length of the tendon was recommended for securing stable ductile behavior.

## Figures and Tables

**Table 1:** Test variables and designation of specimens.

Name	Type of Filler	Type of FRP Surface-Treatment	Number of Tendon (EA)	*f_ck_* (MPa)	Strengthing Length (mm)
Control	-	-	-	40	-
E-OX-1-30-80%	Epoxy	Oxide-coating	1	30	4800
E-OX-1-40-67%	Epoxy	Oxide-coating	1	40	4000
E-OX-1-40-80%	Epoxy	Oxide-coating	1	40	4800
E-OX-1-40-93%	Epoxy	Oxide-coating	1	40	5600

**Table 2:** Physical properties of 10-mm FRP tendon.

Failure Load (kN)	Tensile Strength (MPa)	Median Strain at Rupture (10^−6^)	Elastic Modulus (GPa)
236	3000	17,000	185

**Table 3:** Fatigue test results at upper load limit (Concrete strength).

Beam	Cycle	Deflection (mm)	Change (%)	Strain at Mid-Span					
				Top Steel		Bottom Steel		CFRP Rod	
				Strain (10^−6^)	Change (%)	Strain (10^−6^)	Change (%)	Strain (10^−6^)	Change (%)
Control	1	11.34	-	−181	-	1378	-	N/A ^a^	-
	1000	12.98	14	−187	3	1561	13	N/A	N/A
	2,000,000	14.63	29	−222	23	1820	32	N/A	N/A
E-OX-1-30-80%	1	6.26	-	−212	-	687	-	1135	-
	1000	7.08	13	−194	-8	857	25	1290	14
	2,000,000	11.92	90	−283	33	1088	58	1360	20
E-OX-1-40-80%	1	5.96	-	−242	-	687	-	1001	-
	1000	6.88	15	−251	4	827	20	1167	17
	2,000,000	9.22	55	−274	13	1071	56	1258	26

^a^ Control test specimen is not applied CFRP Rod.

**Table 4:** Experimental results of NSM-strengthened RC beam (Concrete strength).

Beam	Yield Load (kN)	Ultimate Load (kN)	Change (%)	
			Yield Load	Ultimate Load
Control	142.91	183.84	-	-
E-OX-1-30-80%	202.97	277.69	42	51
E-OX-1-40-80%	198.51	276.68	39	51

**Table 5:** Fatigue test results at upper load limit (Bond length).

Beam	Cycle	Deflection (mm)	Change (%)	Strain at Mid-Span					
				Top Steel		Bottom Steel		CFRP Rod	
				Strain (10^−6^)	Change (%)	Strain (10^−6^)	Change (%)	Strain (10^−6^)	Change (%)
Control	1	11.34	-	−181	-	1378	-	N/A ^a^	-
	1000	12.98	14	−187	3	1561	13	N/A	N/A
	2,000,000	14.63	29	−222	23	1820	32	N/A	N/A
E-OX-1-40-67%	1	5.76	-	−195	-	493	-	907	-
	1000	7	22	−197	1	660	34	1084	20
	2,000,000	8.14	41	−253	30	864	75	1225	35
E-OX-1-40-80%	1	5.96	-	−242	-	687	-	1001	-
	1000	6.88	15	−251	4	827	20	1167	17
	2,000,000	9.22	55	−274	13	1071	56	1258	26
E-OX-1-40-93%	1	7.64	-	−230	-	813	-	1053	-
	1000	9.02	18	−221	-4	979	20	1212	15
	2,000,000	11.36	49	−203	-12	1280	57	1397	33

^a^ Control test specimen is not applied CFRP Rod.

**Table 6:** Experimental results of NSM-strengthened RC beam (Bond Length).

Beam	Yield Load (kN)	Ultimate Load (kN)	Change (%)	
			Yield Load (kN)	Ultimate Load (kN)
Control	142.91	183.84	-	-
E-OX-1-40-67%	207.51	283.31	45	54
E-OX-1-40-80%	198.51	276.68	39	51
E-OX-1-40-93%	207.71	283.25	45	54

**Table 7:** Strain gauge location.

Specimen	Strain Gauge Location (mm)		
	Quarter	Center	Quarter
E-OX-1-40-67%	1400	2400	3400
E-OX-1-40-80%	1200	2400	3600
E-OX-1-40-93%	1000	2400	3800
